# Progressive coronary stenosis detected by intraoperative TEE after acute type-A aortic dissection repair: a case report

**DOI:** 10.1186/s40981-025-00802-y

**Published:** 2025-07-01

**Authors:** Asuka Komatsu, Hiroki Tateiwa, Kazumasa Orihashi, Takashi Kawano

**Affiliations:** Department of Anesthesiology and Intensive Care Medicine, Kochi Medical School, Kohasu, Oko-cho, Nankoku, Kochi, 783-8505 Japan

**Keywords:** Acute type-A aortic dissection, Coronary artery stenosis, Transesophageal echocardiography

## Abstract

**Background:**

Acute type-A aortic dissection is a life-threatening condition requiring urgent intervention. Among its complications, coronary malperfusion is particularly fatal. Although rare, coronary artery stenosis after surgical repair is critical yet underrecognized.

**Case presentation:**

A 77-year-old man underwent emergency aortic arch replacement for acute type-A aortic dissection. Intraoperative transesophageal echocardiography (TEE) initially showed no coronary involvement. However, ST-segment elevation and new hypokinesia appeared post-repair. TEE identified progressive left main coronary artery stenosis. Coronary angiography confirmed severe stenosis, leading to urgent coronary artery bypass grafting. The patient recovered well and was discharged on postoperative day 33.

**Conclusions:**

This case highlights the importance of intraoperative TEE for early detection of coronary complications following acute type-A aortic dissection repair. Dissection can progress even after aortic replacement surgery and requires vigilance. Careful monitoring and prompt intervention are crucial to optimize the outcome of these rare but life-threatening events.

## Background

Acute type-A aortic dissection is a life-threatening condition primarily caused by aortic rupture or organ malperfusion, necessitating immediate diagnosis and intervention. Although the operative mortality of acute type-A aortic dissection in Japan has decreased to 9.8% as of 2021 [1], organ malperfusion remains a significant unresolved issue. While modern computed tomography (CT) imaging enables accurate preoperative assessment, malperfusion can still develop intraoperatively. To address this challenge, we have routinely employed transesophageal echocardiography (TEE) during surgical treatment of acute type-A aortic dissection to provide real-time intraoperative hemodynamic and structural assessments. Here, we report a case of acute type-A aortic dissection complicated by the rapid progression of left coronary artery stenosis following weaning from cardiopulmonary bypass (CPB), necessitating urgent coronary revascularization. Notably, TEE allowed for the early detection of structural coronary changes before electrocardiographic (ECG) abnormalities or left ventricular wall motion impairment became apparent, highlighting its potential role in the timely diagnosis and management of intraoperative coronary malperfusion.

## Case presentation

**Fig. 1:**
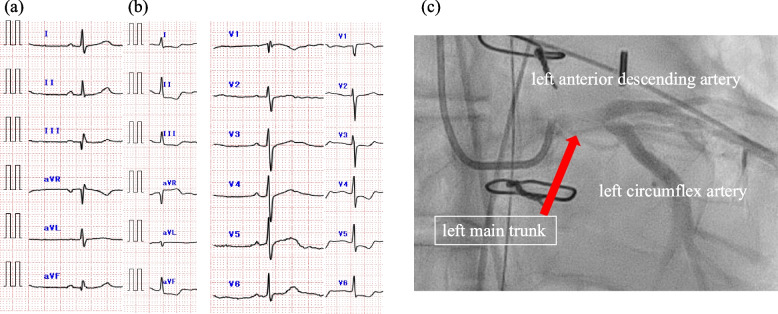
Electrocardiography. **a** Admission electrocardiogram demonstrating no significant abnormalities or ischemic changes. **b** After cardiopulmonary bypass, ST-segment elevation in lead aVR. **c** Coronary angiogram of the left coronary artery, demonstrating occlusion of the left main trunk (arrow)

A 77-year-old man (height;164 cm, weight, 61.7 kg) presented with acute-onset chest pain. His medical history included hypertension and asthma–chronic obstructive pulmonary disease overlap. His vital signs were as follows: heart rate, 56 beats per minute; respiratory rate, 23 breaths per minute; and oxygen saturation, 92% on room air. Brachial blood pressures were 79/51 mmHg (left) and 85/48 mmHg (right). ECG showed no significant abnormalities or ischemic changes (Fig. 1a). Bedside transthoracic echocardiography demonstrated normal left ventricular systolic function and wall motion, with mild aortic regurgitation but no intimal flap or pericardial effusion. CT revealed an acute type-A aortic dissection extending from the aortic root to the level of the renal artery bifurcation, with involvement of the brachiocephalic and left common carotid arteries. The major arterial branches exhibited preserved blood flow. The dissection originated in the ascending aorta. The patient was immediately transferred to the operating room for emergent surgical repair.

**Fig. 2:**
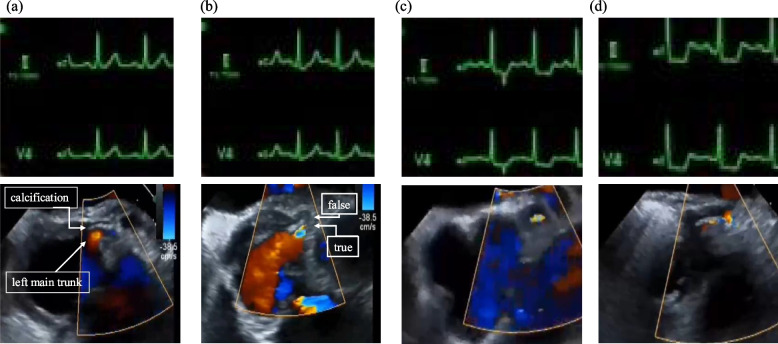
Temporal changes in electrocardiography monitoring and transesophageal echocardiography. **a** Pre CPB, partial calcification was observed at the ostium of the left main trunk (LMT). **b** One hour after CPB, only findings of LMT dissection are observed. **c** Two hours after CPB, ST-segment depression appearing on ECG. **d** Pre CAG, the stenotic ratio reaching nearly 90%

In addition to standard monitoring, electroencephalographic monitor, and brain near-infrared spectroscopy were established. After uneventful rapid sequence induction of general anesthesia with fentanyl, midazolam, and rocuronium, the trachea was intubated. TEE revealed aortic dissection extending proximally to the origin of the left coronary artery and distally to the descending aorta, with the entry site located in the distal ascending aorta. The false lumen was predominantly thrombosed. The dissection extended slightly into the left coronary artery, causing trivial inward displacement of a fine calcified plaque; however, no significant stenosis was noted, and left ventricular function remained normal (Fig. 2a). Based on these findings, ascending aortic replacement was performed. Although CPB was successfully weaned with preserved left ventricular function, TEE performed 1 h later (15:08) revealed progressive displacement of the calcified plaque into the midline of the left coronary artery, despite the absence of ECG changes or left ventricular contraction abnormalities (Fig. 2b). The patient remained hemodynamically stable on dobutamine and nitroglycerin. As this TEE finding was communicated to the surgical team, the coronary artery was closely monitored. The stenotic ratio exceeded 75%, and ST-segment depression emerged 2 h after CPB (15:48), despite stable hemodynamics and preserved wall motion (Fig. 2c). The degree of stenosis was visually estimated based on TEE images. At 2.5 h after CPB, delayed diastolic movement of the anterior left ventricular wall was noted, with the stenotic ratio reaching nearly 90% (18:17) (Fig. 2d). Given these progressive changes, a multidisciplinary discussion involving surgeons, cardiologists, and anesthesiologists was held to determine the appropriate management strategy. It was decided to perform coronary angiography (CAG) to evaluate the severity and extent of stenosis and to guide the choice between percutaneous coronary intervention (PCI) and surgical revascularization. Considering the risks associated with transfer to the catheterization laboratory, intra-aortic balloon pumping (IABP) was initiated after sternal closing (17:30). The operation was completed at (17:43). CAG confirmed > 99% stenosis at the left main trunk (LMT), with no significant stenosis in other coronary segments (Fig. 1c). Based on these findings, surgical revascularization was performed using a left saphenous vein graft to the left anterior descending artery and posterolateral branch artery. Hemodynamic stability was maintained with dobutamine and a low dose of norepinephrine, allowing for IABP removal in the operating room. Postoperative TEE revealed no apparent IABP-related changes in the descending aorta.

The patient was extubated on postoperative day (POD) 1, transferred from the intensive care unit on POD 5, and discharged home on POD 33 after an uneventful recovery.

## Discussion

In this case, TEE played a crucial role in preventing potential hemodynamic collapse in the early postoperative period. We discuss related issues.

First, the patient developed 90% stenosis of the LMT, followed by wall motion abnormalities. We routinely use TEE to monitor three complications of acute type-A aortic dissection—rupture, malperfusion, and aortic regurgitation—throughout key intraoperative phases, starting from post-induction. For malperfusion, we assess the three arch branches and visceral arteries to the extent feasible. Coronary arteries are generally well-visualized, but detection of subtle changes largely depends on vigilance and clinical suspicion. The key parameter in detecting changes was the ratio of the true lumen to the false lumen in the LMT. In this case, calcification at the left coronary ostium served as an important clue. A small change after weaning from CPB caused discomfort, leading to continuous assessment. This highlights the importance of performing TEE assessments immediately after induction of anesthesia as a diagnostic baseline.

Next, progression of coronary artery stenosis following surgical intervention is rare. Coronary artery dissection in acute type-A aortic dissection is associated with high mortality, necessitating prompt intervention [2]. Although no studies have examined intraoperative extension, similar cases likely share poor prognoses. In aortic dissection, pathological findings reveal disruption of the intimal bridging structures [2], increasing the risk of iatrogenic dissection due to suturing or other surgical manipulations [3, 4]. Coronary artery dissection occurs in 0.07% of catheter-based coronary interventions, and selective cardioplegia delivery protection during cardiac surgery may also contribute [5, 6]. In this case, TEE confirmed the absence of flap injury in the LMT at the time of CPB weaning, after cannula insertion. This suggests that the dissection likely resulted from the intimal cutting caused by suturing during proximal anastomosis, which may have created a micro-entry. The subsequent increase in false lumen pressure may have led to compression and narrowing the true lumen of the LMT. Currently, predicting the occurrence and timing of such events remains exceedingly challenging. The most reliable approach is a precision stepwise assessment, with TEE serving as an optimal tool for this purpose.

**Fig. 3:**
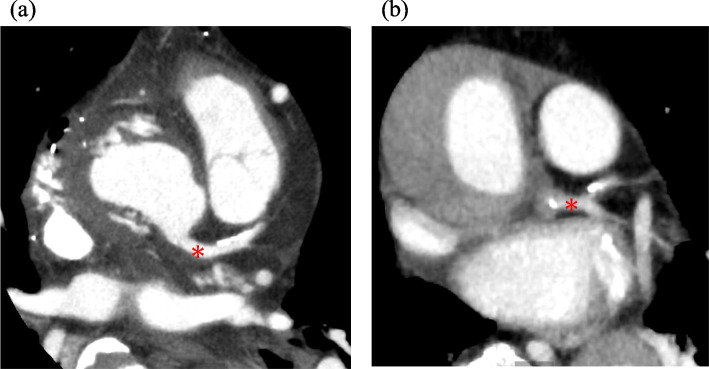
Computed tomography. **a** Preoperative and **b** postoperative left main trunk (LMT) (*). The dissection of the LMT has resolved

Finaly, the treatment approach is presented. Some institutions may opt for coronary artery bypass grafting (CABG) based on TEE findings alone. However, we had no prior experience with intraoperative coronary dissection occurring in this progressive manner, nor had we encountered TEE findings of coronary dissection other than cases of static lesions in aortic valve replacement. Therefore, following multidisciplinary discussion, we elected to perform CAG. Given the risks of patient transfer to the catheterization laboratory, an IABP was inserted, though opinions on this decision may vary. While IABP is generally contraindicated in acute type-A aortic dissection due to risks of aortic expansion or rupture, prior reports have described its successful use for hemodynamic stabilization without these complications [7]. Furthermore, intraoperative TEE confirmed that the guidewire was accurately placed into the true lumen. Regarding treatment selection, both PCI and CABG were considered. Although PCI for LMT stenosis due to aortic dissection has been reported [8, 9], the stenosis extended proximally to both the left anterior descending and left circumflex arteries in this case. Thus, CABG was selected to avoid direct intervention at the affected site. Postoperative contrast-enhanced CT revealed regression of the false lumen, likely due to increased true lumen pressure following bypass (Fig. 3). In retrospect, in cases with sufficiently clear intraoperative TEE findings, immediate surgical revascularization without angiography may be a reasonable approach.

In conclusion, this case shows the diagnostic and clinical utility of intraoperative TEE in detecting coronary stenosis after acute type-A aortic dissection repair. The progressive and vigilant monitoring of TEE is imperative for the timely diagnosis and implementation of appropriate interventional strategies.

## Data Availability

Please contact the corresponding author for data requests.

## Abbreviations

TEE: Transesophageal echocardiography
CT: Computed tomography imaging
CPB: Cardiopulmonary bypass
ECG: The 12-lead electrocardiogram
CAG: Coronary angiography
PCI: Percutaneous coronary intervention
IABP: Intra-aortic balloon pump
LMT: Left main trunk
POD: Postoperative day
CABG: Coronary artery bypass grafting
